# Enhancement strategies for mesenchymal stem cells and related therapies

**DOI:** 10.1186/s13287-022-02747-w

**Published:** 2022-02-21

**Authors:** Senthilkumar Alagesan, Jack Brady, Declan Byrnes, Juan Fandiño, Claire Masterson, Sean McCarthy, John Laffey, Daniel O’Toole

**Affiliations:** 1 Discipline of Anaesthesia, National University of Ireland, Galway, Ireland; 2 Discipline of Anaesthesia, Galway University Hospital, Galway, Ireland

**Keywords:** Stem cells, Priming, Cell therapy, Extracellular vesicles

## Abstract

Cell therapy, particularly mesenchymal stem/stromal (MSC) therapy, has been investigated for a wide variety of disease indications, particularly those with inflammatory pathologies. However, recently it has become evident that the MSC is far from a panacea. In this review we will look at current and future strategies that might overcome limitations in efficacy. Many of these take their inspiration from stem cell niche and the mechanism of MSC action in response to the injury microenvironment, or from previous gene therapy work which can now benefit from the added longevity and targeting ability of a live cell vector. We will also explore the nascent field of extracellular vesicle therapy and how we are already seeing enhancement protocols for this exciting new drug. These enhanced MSCs will lead the way in more difficult to treat diseases and restore potency where donors or manufacturing practicalities lead to diminished MSC effect.

## Introduction

It is almost impossible to catalogue all of the therapeutic investigations conducted and ongoing using stem cells. However, the excitement in this burgeoning field has been somewhat dampened by some less than stellar clinical trial results and persistent variability of effect due to production practicalities and correlative, rather than causative, potency assays. During these studies an immense amount of data has been generated regarding stem cell biology and possible mechanism of action in diseases, including the physiological niche where various stem cells are sourced, cell–cell contact-dependent mechanisms and a rich secretome containing small molecules, proteins, organelles and even full membrane bound bodies. Indeed, much of this data has been accumulated regardless of whether the overall subsequent clinical trials themselves were successful. These have prompted a multitude of strategies that could putatively be included in the cell manufacturing process to improve outcomes in patients. In this review we look at the more important of these strategies to give the reader an insight into the next generation of stem cell and stem cell-derived therapies.

## MSCs as drug delivery vectors

Often, in vivo delivery of therapeutic agents is hampered by obstacles such as their short half-life, poor solubility index, clearance shortly after administration, as well as a low targeting ability and potential toxicity toward healthy tissues. Therefore, many pharmacological, natural, and biological-based therapeutics require transport, protection, and direction toward the target site in vivo to improve their therapeutic index.

There are several distinct advantages in using MSCs as drug delivery vectors, many of which are part of their appeal as a therapeutic in unmodified form; they are biologically derived, circumventing many of the concerns associated with using chemically derived particles, and have a proven safety profile in patients [1]**.** MSCs exhibit intrinsic therapeutic abilities in a range of injuries and diseases [2] which would serve toward a potential adjunct therapeutic strategy with a drug of interest. They are immune-evasive with the ability to avoid detection and clearance by the host immune system due to a lack of major histocompatibility complex (MHC)-II and low MHC-I expression on the cell surface [3] which would provide the ideal carrier system for therapeutics which are rapidly targeted and cleared or invoke an unwanted immunological response. MSCs are also known to home to sites of injury and inflammation [4] and can also be administered in vivo via several different routes including systemic injection, direct injection, aerosolization, and topical administration [5]. Finally, they can be easily isolated, cultured and expanded, with the potential for ex vivo modification prior to administration, allowing the use of several methods of drug loading each with their own advantages and disadvantages.

### Passive drug loading

The principle of passive drug loading of cells rests mainly in the concentration gradient between the cell cytoplasm and the solution containing the therapeutic of interest into which the cells are submerged. Conveniently, the cytosol will often become equiosmotic with the extracellular environment when immersed in certain drug solutions enabling straightforward drug loading processes. However, this will depend on the properties of the drug in question—its hydrophobic status will impact on uptake in this manner. For example, Paclitaxel, docetaxel, camptothecin, and etoposide are lipophilic chemotherapeutic drugs which can be loaded into MSCs via simple diffusion, proven to be an effective drug loading strategy (reviewed in [6]). However, if a cell can be passively loaded along a concentration gradient, it could also potentially ‘unload’ after in vivo cell administration at the incorrect site and so efficient administration techniques must be considered. In turn, the concentration taken up by the MSC may not be sufficient to have a therapeutic effect at the site of injury but could however impact the cell itself. Rigorous ex vivo testing is needed to confirm the suitability of a drug for passive loading to MSCs.

### Cell membrane protein-mediated drug loading

All cell membranes host a variety of proteins and ligands which can be a focus for drug loading. Broadly speaking, these cell surface entities either bind or transport, and in MSCs this is no different as they host a range of structures investigated for drug loading and delivery. Human concentrative nucleoside transporter 1 (hCNT1) and human equilibrative nucleoside transporter 1 (hENT1) are present in high levels in MSCs and are the main transporters of the chemotherapeutic drug gemcitabine in ovarian cancers [7]. This has been exploited for use in MSC-mediated delivery of chemotherapeutics to treat certain cancers [8, 9]. The expression of P-glycoprotein (P-gp) transporters has been shown in MSCs [10], and the chemotherapeutic paclitaxel which binds P-gp has been demonstrated to be an effective drug to load into MSCs with sufficient uptake and release profiles as well as minimal effect on the vector MSC [11]. The biological processes of MSCs can be exploited to effectively load a drug of interest into the cell. By coating the therapeutic (or nanoparticles loaded with therapeutic) with MSC receptor ligands, binding to the cell surface will occur. MSC endocytosis is mediated via scavenger receptors such as macrophage receptor with collagenous structure (MARCO) and scavenger receptor class B type 1 (SR-B1), and the mannose receptor cell determinant (CD)206 [12] allowing for targeted uptake of drugs. The process of cell exocytosis can also be utilised to facilitate drug unloading [13].

### Physical/chemical drug loading

MSCs can be manipulated using methods such as electroporation, transfection, and microinjection to directly load the cells with the drug. This has several advantages as these are tried and tested methods with overall good results in other areas such as genetic modification. However, in terms of drug loading this has not been thoroughly investigated and several issues are apparent. The cells are generally not given time to ‘rest’ following loading with therapeutic due to its half-life (resulting in unpredictable dosing), and intracellular effects of the drug which may affect cell viability and function unpredictably. Resting, therefore, may not be an option when drug loading using chemical or physical mechanisms, as it is often required to ascertain efficacy and cell viability. Cells may be compromised due to the aggressive nature of the strategies used which would outweigh the benefit of loading them with therapeutic drugs. Liposome-based transfection is probably the most widely used method to load substances into cells. It is most commonly used to deliver genetic material however, which is discussed elsewhere in this review.

### Cell surface drug loading

Another strategy to use MSCs to deliver therapeutic drugs is to coat the surface of the cell. Several characteristics of MSCs make this a viable option: firstly, the cells are negatively charged and will attract and attach positively charged molecules; secondly the hydrophobicity of cells, including MSCs, influences the adhesion of a range of molecules including bacteria [14] and membrane bound carriers. The avidin–biotin complex (ABC) method of cell-drug interaction involves the use of biotin which binds to the MSC surface and will then rapidly and stably form a covalent bond with an avidin-coated particle (reviewed in [15]). Alternatively, the MSC can be ‘avidinated’ followed by binding of biotinylated drug molecules [16] and liposomes [17] (Table 1).

**Table 1:** Comparison of drug loading approaches

Method	Advantages	Disadvantages
Passive Loading	Simple method	Drug can alter cell properties
	Uses concentration gradient	Passive unloading
		Not suitable for hydrophobic drugs
Receptor mediated loading	Direct targeting toward receptor	May require use of nanoparticles
		Expensive ligands
Physical loading	Tried and tested methods	Invasive
		Impacts cell viability
Liposome-mediated Loading	Well researched	Expensive method when scaled up
	Can be enhanced by targeting receptors/surface modification	
	Protects the delivered drug	
EV vector loading	Naïve MSC EVs are therapeutic	Not fully characterised
	Easily isolated	Poor targeting in some diseases
	Proven to carry cargo from parent cell	Can be difficult to obtain large numbers

### MSC extracellular vesicles

The use of biological particles to deliver drugs to cells is a promising avenue of research which overcomes many of the disadvantages incurred in the methods discussed so far. Utilizing membrane bound vesicle to harbour therapeutics prevents the loaded cell from reacting to drug exposure, reduces the possibility of passive unloading of the drug, can circumvent the problems with hydrophobic drugs (lipid core), provides a surface for ligand coating and can be delivered by non-invasive means to a cell. In turn there are many promising liposome carriers that are not entirely suitable for systemic delivery or that have not shown efficacy [18] and would benefit from a cell vector. Not only would MSCs be a promising drug delivery vehicle, but the extracellular vesicles (EVs) produced by MSCs have also been shown to have therapeutic properties akin to their parent cells [19] and in fact demonstrate several distinct advantages for clinical translation [20, 21]. There is also the capacity to load these EVs by manipulation of the parent cell, a means by which drug-containing EVs can be produced with a greater loading capacity than commercial liposomes [22]. This method is especially suitable for molecules and proteins which are incorporated into lipid rafts, which are often contained in EVs and exosomes [23]. As well as the capacity for MSCs to produce EVs loaded with therapeutics and drugs they are exposed to, MSCs can also take up nanoparticles coated with/containing the drug of interest, and create EVs containing these [24].

### Limitations and future directions

The knowledge we have already gained in the use of MSCs as a therapeutic strategy shows that the possibilities are, as yet, not fully elucidated (See Table 1 for summary of current approaches). The MSC’s morphological and phenotypical changes in response to any kind of exogenous manipulation highlights that these cells can be further enhanced to produce a powerful therapeutic tool with the potential for use in a huge number of diseases and injuries. What we know from studies on other cell types as drug delivery vectors will inform toward the methodology to be applied to MSCs [25]. In terms of receptor mediated drug loading there are also several opportunities available, as previously examined in other cells. For example, the transferrin receptor has been targeted for drug uptake in vivo by conjugating drugs and liposomes with transferrin [26]. The role of the transferrin receptor in MSCs has been examined of late [27], with the observation that excess iron can affect MSC function, observed where magnetic iron oxide particles were used for MSC tracking studies and homing was influenced by expression of chemokine receptor type 4 (CXCR4) [28]. Glucose transporters (GLUT) have the potential to uptake molecules associated with polysaccharides, a process known as glycoconjugation. MSCs express GLUT receptors which can be upregulated in response to exposure to hypoxia, increasing their glucose uptake capacity [29]. MSC folate receptors have been targeted for uptake of genetic material [30] and could potentially be repurposed for the delivery of folic acid conjugated drug molecules. In essence, there are several different receptors and transporters expressed by MSCs which could be examined for drug loading and delivery strategies.

Naïve MSCs as an adjunct therapy is an exciting avenue of research that is ongoing in several research areas [31–33] with the potential for drug loaded MSCs to further the prospects of the development of a highly effective therapy.

As the full mechanism of action and properties of MSCs in response to different priming conditions have not been fully revealed, it is not yet possible to determine the full effect of drug loading on the behaviour of the cell. It is also worth remembering that stem cell longevity after transplant is generally poor (indeed death of the cell therapy may be required for efficacy in certain disease contexts [34, 35]) and thus drug loading would be a poor approach to long term therapy. However, results thus far have been promising, for example the fact that MSCs are resistant to the cytotoxicity of chemotherapeutics such as paclitaxel. However, herein lies another limitation—the majority of MSC drug loading techniques have been geared toward effective chemotherapeutic delivery and as yet other conditions and injuries are poorly studied.

## Enhancing direct anti-microbial properties of MSCs

While there is plenty of evidence to support the fact that MSCs provide an anti-microbial effect, modulating the immune system and secreting anti-microbial peptides (AMP) to directly interfere with pathogens, not many studies investigate different licensing strategies to enhance the release of AMPs. As reviewed by Byrnes et al. [36], AMPs function by disrupting pathogen membranes and replication while also acting as a chemoattractant to recruit immune cells to the site of infection. To date, MSCs are known to express The cathelicidin antimicrobial peptide (LL-37), human β-defensin-2, hepcidin, and lipocalin-2 which target bacteria, viruses, parasites, and fungi through direct and indirect mechanisms [36].

One of the earliest licensing strategies was exposing MSCs to live bacteria [37–39]. It was shown that BM-MSCs in the presence of *E. coli* significantly increased the production of LL-37, this was further confirmed to be the mechanism of action in reducing bronchoalveolar lavage (BAL) bacterial counts in an animal model of *E. coli* pneumonia [39]. Sung et al. [37] showed that MSCs significantly upregulated β-defensin 2 upon exposure to *E. coli* and this effect was not seen in fibroblasts. However, a bacterial mix of mouse faeces did not induce detectable levels of LL-37 or β-defensins [1–3] but did significantly inhibit bacterial growth. This was found to be due to an increase of hepcidin by up to 50-fold; with this effect being lost under hypoxic conditions, highlighting the importance of culture conditions for MSC efficacy [38]. Taken together, these papers show large levels of donor-to-donor and bacterial stimulator source variability in the secretion of AMPs by MSCs.

Even though the majority of papers utilise live bacteria to enhance AMP production in MSCs, others have shown that pathogen-associated molecular patterns (PAMPs) [40, 41] and cytokine licensing [42, 43] also amplifies their expression. It was found that the endotoxin lipopolysaccharide (LPS) enhanced the production of lipocalin 2, and this effect was dramatically increased by co-stimulation with tumor necrosis factor (TNF)-α. In their animal model of *E. coli* pneumonia, they showed significantly reduced *E. coli* in the bronchoalveolar lavage (BAL) and significantly increased lipocalin 2 with intratracheal administration of MSCs [44]. In subsequent in vitro experiments, the levels of lipocalin 2 were postulated to be due to alveolar macrophages secreting TNF-α in response to the infection, thereby activating the naïve MSCs administered intratracheally to produce lipocalin 2 [41]. Sutton et al. [43] further showed the effectiveness of MSCs by licensing them with interferon-γ (IFN-γ), interleukin-1β (IL-1β), or interleukin-12 (IL-12) which increased the secretion of LL-37, significantly reducing the rate of growth in *P. aeruginosa*, *S. aureus*, and *S. pneumonia*. The MSCs also enhanced antibiotic sensitivity in these bacterial strains emphasising their potential as a conjunctive therapy in infections.

Of remarkable interest is the potential of these AMPs from MSC conditioned media (CM) as a treatment method. McCarthy et al. [45] showed that nebulised MSC-CM significantly reduced the proliferation capabilities of *E. coli*, *K. pneumoniae*, and *S. aureus*. While both hepcidin and lipocalin-2 passed through the nebuliser intact, LL-37 was undetectable post-nebulisation, despite antibacterial effects being apparently unchanged. This indicates the potential therapeutic efficacy for administrating MSC-CM to patients with bacterial pneumonia and along with conventional anti-biotic treatments these could be an effective treatment method for resistant strains of bacteria.

## Licensing strategies to enhance MSC therapeutic effects

Recently this topic has been reviewed by Byrnes et al. [36] who laid out different licensing strategies for MSCs in tabular format in the context of sepsis and ARDS. Since there is a multitude of papers on potential licensing strategies to enhance MSC function, for this analysis the focus will be on selected papers that elucidate upon the mechanism of action for their activated MSCs.

Interestingly one of the only papers targeting fungal infections in a unique manner is with the selective isolation of a subset of IL-17^+^ MSCs. They elicit a significant reduction in the growth of *C. albicans* in a colitis animal model compared to IL-17^−^ and mixed MSCs. The therapeutic efficacy was found to be directly due to the production of IL-17, leading to restored kidney structure. However, the trade-off is a reduced immunomodulatory effect with a reduction in T-regulatory cells due to impaired TGF-β1 secretion, which again was directly due to the production of IL-17. This emphasises the importance of targeted strategies for the different licensing strategies of MSCs [46]. IL-17 plays a critical role in defence against fungal infections [47].

Keeping to the theme of the potential for MSCs to treat infections, an interesting study by Mesiel et al. [42] demonstrated the effectiveness of licensing MSCs with IFN-γ, alone or in combination with either IL-1β, or TNF-α to enhance the secretion of indoleamine 2,3-dioxygenase (IDO). While IDO is known to be a potent immunomodulator against T, B, and NK-cells they found that IFN-γ or TNF-α were potent enhancers of IDO which was an underlying mechanism for reducing the proliferation of a wide range of clinically relevant bacteria, parasites, and viruses in direct co-cultures with MSCs. This finding that IFN-γ enhanced IDO secretion was recently confirmed by Boyt et al. [48] who showed this across three different donors. They also noted that dose and duration of IFN-γ should be tailored to the individual donor to maximise IDO secretion emphasising that donor-to-donor variability plays an important role when discussing the efficacy of MSCs.

As shown above, one of the most prominent licensing strategies is a combination of cytokines, known as ‘cytomix’. Typically, this includes one or all of the pro-inflammatory cytokines TNF-α, IL-1β, and IFN-γ potentially in combination with other stimulants to drastically enhance the efficacy of the MSC. This has already been shown to be effective in an animal model of VILI with TNF-α, IL-1β, and IFN-γ licensed MSCs restoring oxygenation, reducing inflammatory cytokines and neutrophils in the BAL fluid, improving lung compliance, preventing leaky capillaries, and improving lung structural injury. This was found to be in part due to KGF secretion by licensed MSCs, enhancing epithelial wound repair [49].

## Hypoxia priming

Standard cell culture practice generally uses normoxic oxygen tension which is that of atmospheric pressure (21% O_2_). Hypoxia in the context of cell culture refers to oxygen tensions ranging from 0 to 10% [50]. It is worth noting that physiological oxygen tension in tissues can be detected as low as 1% in cartilage and bone marrow (BM), and up to 12% in peripheral blood, which is still much lower than the 21% O_2_ used routinely to culture MSCs [51]. For this reason, along with the fact that MSCs themselves are naturally located in hypoxic environments within the body [52], it is believed that pre-conditioning MSCs in this way could be largely beneficial to their therapeutic use. MSCs are capable of switching from aerobic to anaerobic mechanisms allowing them to exist comfortably in these low oxygen environments [53] and do this in part through the upregulation hypoxia inducible factor (HIF)-1α which is known to promote hypoxia tolerance [54].

Hypoxia used as a pre-conditioning tool has been shown to be quite effective across a wide range of disease conditions by improving MSC cell survival, downregulating apoptosis related pathways [55, 56], enhancing MSC pro-survival markers, enhancing MSC release of chemoattractant and growth factors involved in cell proliferation, enhancing MSC anti-oxidant effects and by enhancing MSC related angiogenesis [57–59]. Hypoxia-primed MSCs have also been shown to have higher levels of glucose consumption, reduced production capacity of reactive oxygen species (ROS) and have lower telomeric shortening rates leading to decreased cellular senescence [60].

Hypoxia pre-conditioning of MSCs in relation to lung specific disease has shown many benefits. MSCs cultured under hypoxic conditions (1% O_2_) were shown to successfully attenuate ischemia/reperfusion (I/R) pathologic lung injury score by inhibiting inflammatory responses associated with ROS generation and also demonstrated anti-apoptotic effects [61]. The therapeutic effects in this study were only observed at low dose administration (2.5 × 10^5^ cells) which resulted in the down-regulation of P38 mitogen-associated protein kinase (MAPK) and nuclear factor (NF)-κB signalling and upregulation of glutathione, prostaglandin E2, IL-10, mitochondrial cytochrome c and B-cell lymphoma (Bcl)-2 [61]. Low dose administration also showed cell migration into interstitial alveolar spaces and bronchial trees, while high dose administration (1 × 10^6^ cells) caused cell aggregation in the microcirculation and caused pulmonary embolism [61].

The therapeutic effect of MSCs for radiation-induced lung injury (RILI) has been shown to be limited because of local hypoxia levels and extensive ROS in irradiated lungs [62]. Since this was considered to mainly be due to the poor survival of MSCs, Li et al. hypothesised that persistent and adaptive hypoxia pre-treatment of bone marrow MSCs prior to their transplantation in injured mice would enhance their survival and improve their therapeutic effect [58]. Comparing normoxic (21%) or hypoxic (2.5%) cultured MSCs, the researchers found that the hypoxic MSCs had higher cell viability, enhanced proliferation and improved HIF-1α mediated anti-oxidant ability, and overall enhanced therapeutic efficacy against RILI [58].

It has been well described that levels of engrafted MSCs are dramatically reduced after 24 h of transplantation as a result of the toxic and oxidative microenvironments to which they are introduced [63]. In a mouse model of bleomycin-induced pulmonary fibrosis, pre-conditioning MSCs with hypoxia enhanced the survival rate of engrafted MSCs which in part was due to the upregulation of hepatocyte growth factor [64]. This group also demonstrated that hypoxia-cultured MSCs attenuated extracellular matrix production through paracrine effects in transforming growth factor (TGF)-β1-treated MRC-5 fibroblast cells [64].

In a rodent model of ventilator-induced lung injury, MSCs cultured in hypoxic (2%) conditions in combination with cytokine pre-activation successfully decreased stretch-induced pulmonary epithelial inflammation and injury, restored oxygenation, improved lung compliance and reduced lung leak with improved resolution of lung structural injury [49].

Wang et al. recently reported beneficial effects of hypoxia pre-treatment of BM MSCs. Combining this with curcumin, a natural dietary product with known protective effects on various cellular processes they reported an improvement in cell survival, an enrichment in cells in the G2/M and S phase of the cell cycle and improved mitochondrial function in the BM MSCs [65]. They also demonstrated a strong reduction in cytochrome c release from the mitochondria with a subsequent decrease in caspase-3 cleavage and a suppression of apoptosis. Moreover, mitochondrial quality was enhanced as a result of increased mitochondrial fusion and elevated activity of oxidative phosphorylation and mitochondrial complex 1 enzymes in the MSCs [65]. Lastly hypoxia pre-conditioning in combination with curcumin significantly enhanced wound healing in a mouse model of wound closure [66].

## Enhancement of effect through differentiation prior to engraftment

Priming of MSCs using biochemical and biophysical mechanisms has been studied and shown to directly affect the stem cell fate towards specific phenotypes. These strategies aim to modulate the environment through the use of different biomaterial surfaces of either natural or synthetic sources and also through the use of various culture conditions [67, 68]. Tissue engineering strategies have been utilised to direct MSC differentiation towards desired phenotypes using 3D-modelled scaffolds for cell culture. Creating these micro-environments offers structural as well as biochemical support for MSCs which can enhance tissue healing [69]. Additionally, these biomaterial-based approaches can play a beneficial role in avoiding cell death due to anoikis and/or inflammation [70].

There are many factors which need to be understood to fully direct stem cell fate. These include relative stiffness of the culture material, topography, geometry and chemical composition [71]. The effects of matrix stiffness on MSC differentiation capacity was first described in 2006 by Engler et al. [72]. In this study, it was described that the elasticity of different matrices modulate MSC differentiation and result in distinct MSC phenotypes [72]. In brief, it was observed that cells grown on soft substrates (elastic modulus, 0.1–1 kPa) began to resemble cells of a neural lineage, where as those grown on stiff (8–17 kPa) and harder (24–40 kPa) surfaces resembled those of myogenic and osteogenic lineages respectively [72].

Wu et al. showed that not only were biochemical cues important to mimic a cellular microenvironment, but also physical features including nano-topographical features for cell/matrix interaction [73, 74]. The group showed that various topographical patterns induced changes in MSC morphology and cytoskeleton structure which affected cell aggregation and supported differentiation towards the chondrogenic lineage [73, 74]. The importance of these nano-topographical features can also be seen in the different surfaces that that can maintain MSC multipotency or drive the cell towards osteogenic differentiation. Using near identical culture materials in terms of chemistry, stiffness and physical properties, it has been observed that off-setting the centre position between pits by ± 50 nm is enough to change the fate of the cell [75].

Raic et al. developed a fibrous scaffold that resembled bone/bone marrow extracellular matrix based on protein without the addition of synthetic polymers. The authors report that the fibrous structures used, as well as the charge of the material, were the key contributors to MSC differentiation [76]. Priming MSCs on soft matrices has been shown to improve wound healing over MSCs primed on stiffer matrices [77].

Specifically primed MSCs have emerged as a useful therapeutic strategy in the area of bone tissue engineering. Culture medium consisting of dexamethasone, ascorbic acid 2-phosphate (AsAP), and β-glycerophosphate has shown to improve levels of calcium matrix deposition and enhance the expression of osteogenic markers in MSCs. In this study, cells grown on collagen/hydroxyapatite material showed the best osteogenic capacity [78]. For cartilage repair, MSCs primed in chondrogenic medium and encapsulated in a methacrylated hyaluronic acid scaffold and treated with dynamic loading in a bioreactor showed superior chondrogenic differentiation and survivability compared to untreated MSCs [79]. Additionally, the authors reported significantly improved neocartilage formation in the treated MSC group in a rat model of osteochondral defect [79]. Lam et al. [80] similarly reported enhanced cartilage regeneration in chondrogenically and osteogenically pre-differentiated MSCs.

Glucose levels in MSC culture medium have also been shown to impact MSC differentiation capacity. It has been shown that high-glucose medium facilitates osteogenic differentiation much better when compared to culture medium containing lower glucose levels [81]. Furthermore, high-glucose culture medium prior to chondrogenic differentiation has been show to hinder its chondrogenic capacity, which has been shown to occur through its effects on protein kinase C and TGF-β receptor [82]. These findings suggest that culture in low glucose conditions is required for chondrogenic differentiation, but also have implications in patients with hyperglycaemia [82].

MSC therapeutic potential has been shown to be enhanced with radiation induced activation. Stimulating cells with 2 Gy low-energy transfer ionizing radiation has shown significant differences in the proteins contained in secreted exosomes versus the protein profile observed in non-stimulated MSCs [83]. The authors report on key components of cell–cell or cell–matrix adhesion, including annexins and integrins, and link it with enhanced tumor cell death. It was also noted that the amount of protein present in pre-irradiated cells was 1.5 times greater compared to nonirradiated controls and this finding correlated with enhanced anti-tumor activity when combined with radiation therapy [84].

## Gene modified MSCs: survival, secretion, homing

During the last decade, genetic modification of mesenchymal stromal cells by using viral and non-viral methods has been developed for increasing therapeutic properties and survival of MSCs. Genetic modification of MSCs has several advantages in the development of therapeutic approaches in oncology and regenerative medicine, among others. In this section we are going to discuss the state of the art of the genetic engineering for modifying MSCs to enhance their therapeutic properties.

MSCs have several advantages for use as an antitumour therapeutic: they are naturally immune privileged, they have a trophism and homing properties into solid tumours, and they are not inhibited by suppressive tumour microenvironments. Due to these characteristics, genetic modification of MSCs for expressing certain proteins of interest is a good therapeutic strategy for tumour malignancies. The overexpression of TNF-related apoptosis-inducing ligand (TRAIL) in adipose tissue-derived mesenchymal stromal cells has inhibitory properties on in vitro H460 tumour growth and in vivo after subcutaneous injection on an H460 xenograft model [85]. Dodecameric TRAIL (dTRAIL) can also be overexpressed with herpes simplex virus thymidine kinase (HSV-TK) in MSCs for the treatment of de-differentiated liposarcomas lung metastasis. Jo and colleagues have demonstrated that a single dose of toxic ganciclovir after the intravenous (IV) administration of these genetic modified cells eliminates lung nodules in an animal model of lung metastasis; and this effect is even greater with a double injection of these genetically modified cells [86]. Another interesting strategy for the treatment of solid tumours is cytokine overexpression into the tumour microenvironment to induce an immune-mediated antitumour response and immune memory. Gonzalez-Junca and colleagues have developed genetic modified BM-MSCs that express both IL-12 and IL-21, named as SENTI-101 [87]. They demonstrated that intraperitoneal administration of SENTI-101 in an animal model of peritoneal carcinomatosis increases both innate and adaptive immune responses and increases the T-cell memory population [87]. The genetic modification of MSCs for cancer therapy can also be used for the cross-priming of other cells. BM-MSCs genetically modified for expressing IL-7 and IL-12, in comparison to normal BM-MSCs, increased the antitumour response of chimeric antigen receptor (CAR)-T cells in an animal model of colorectal cancer [88]. Genetically modified MSCs are also relevant as a therapeutic and/or prophylactic vaccination strategy against cancer. MSCs genetically modified for expressing the thymoproteasome complex can induce a potent T-cell immunity against cancer as the result of cross-priming endogenous dendritic cells by presenting cancer antigens [89].

Other areas where genetic modifications of MSCs is interesting is in the development of different tissues for regenerative medicine. Weißenberger and colleagues have generated different types of cartilage neotissue in collagen hydrogels [90]. They developed genetically modified BM-MSCs with one of each three different genes: sex-determining region Y-type high-mobility-group box 9 (SOX9), transforming growth factor beta 1 (TGFB1) or bone morphogenetic protein 2 (BMP2) and obtained cartilage tissues with a different degree of hypertrophy depending on the gene used [90]. Park and colleagues have used genetically modified BM-MSCs expressing hepatocyte growth factor that they have encapsulated in an epicardially implanted 3D scaffold in an animal model of myocardial infarction [91]. These genetically modified MSCs improved vasculogenic potential and cell viability, which ultimately enhanced vascular regeneration and restored cardiac function [91]. Genetically modified MSCs can be also used for restoring gene expression in certain genetic diseases caused by loss of function mutations. Petrova and colleagues designed a genetically modified MSC for expressing collagen type VII alpha 1 chain (COL7A1) for the treatment of recessive dystrophic epidermolysis bullosa (RDEB) [92]. When injected intradermally in a skin patch of an RDEB patient grafted in a mouse, they restored normal levels of tissue COL7A1 and recovery of normal function; but no effects were observed when the MSCs were administered intravenously [87].

Finally, genetic modifications can also induce a potent anti-inflammatory response on MSCs, directly, or indirectly. Genetically modified adipose MSCs transiently co-expressing IL-10 and chemokine (C-X-C motif) ligand 4 (CXCL4) enhanced in vivo homing to inflamed areas and their anti-inflammatory effect, in comparison to unmodified MSCs [93]. On the other hand, MSCs that transiently express the cytokine IL-4 can induce a strong polarization of macrophages toward the anti-inflammatory M2 phenotype in an animal model of traumatic brain injury, despite no improvement in outcomes [94]. More studies in other inflammatory diseases should be done to investigate the strong polarization of macrophages by these genetically modified MSCs.

## MSC priming with pharmacological drugs and small molecules

In previous sections, we have detailed some of strategies which have been used in the field to enhance the therapeutic efficacy of MSCs. In this section, we have reviewed, the benefits of MSC priming via pharmacological drugs and small molecules. To this end, we have reviewed 14 relevant peer reviewed research articles to summarise the effects of pharmacological drugs and small molecules on MSCs both in vitro and in vivo (please see Table 2). Priming of MSCs with pharmacological drugs and small molecules has shown promising outcomes in various disease models [95–101]. A range of pharmacological drugs and small molecules have been used to prime MSCs to enhance their therapeutic benefits via altering their immune-modulatory properties, survival, homing, mobilisation and engraftment. Valproic acid in combination with either lithium or sphingosine-1-phosphate, all-trans retinoic acid (ATRA), 2,4-dinitrophenol (DNP) and b3 adrenergic agonists (b3AR) in combination with CXCR4 antagonist are shown to augment the homing, mobilisation and engraftment of MSCs [95, 98, 99, 102, 103]. A study by Lim et al. [103] has shown that low doses of valproic acid in combination with sphingosine-1-phosphate pre-treatment upregulated genes associated with stem cell migration and anti-inflammatory response. In another study by Linares et al. [95], intra-nasal delivery of MSCs pre-treated with valproic acid in combination with lithium is shown to possess superior therapeutic benefits in a mouse model of Huntington’s disease. The therapeutic benefits of MSCs pre-conditioned with valproic acid and lithium were attributed to enhanced upregulation of genes associated with trophic factors, anti-oxidants, anti-apoptosis, mitochondrial bioenergetics and stress response pathways [95]. Priming of MSCs with all-trans retinoic acid (ATRA) enhanced survival and engraftment after administration [98, 99]. ATRA-primed MSCs are shown to significantly reduce T-helper-17 (Th-17) and T-reg cells in in vitro co-culture assays with peripheral blood mononuclear cell (PBMC) samples from ankylosing spondylitis patients. Cytokine analysis from the co-culture assays showed significant reduction of disease progressing cytokines such as TNF-α, IL-17α and IFN-γ [102]. In another study by Pourjafar et al. [99] ATRA treated MSCs were shown to be better both in in vitro and in in vivo rat wound healing models. In vitro ATRA treated MSCs had increased expression of cell survival and growth factors such as cyclooxygenase (COX)-2, HIF-1α, CXCR4, C–C motif chemokine receptor (CCR)-2, vascular endothelial growth factor (VEGF), angiopoietin (Ang)-2 and Ang-4. In the same study, an in vivo rat wound healing model showed that ATRA primed MSCs were superior in wound closure with improved angiogenesis in comparison to control MSCs [99]. ATRA are also used to prime MSCs to enhance their regenerative properties. In the mouse model of elastase induced emphysema, ATRA and MSC combinations are shown to be more effective in improving static lung compliance, mean linear intercepts and alveolar surface area in comparison to control groups. The therapeutic benefits of ATRA and MSCs were attributed to activation of P70S6 Kinase-1 in MSCs. P70S6 Kinase-1 overexpressing MSCs plus ATRA was shown to be even more beneficial in vivo when compared to MSCs and ATRA [98].

**Table 2:** Small molecule enhancers of MSC efficacy

Pharmacological/small molecule	In vitro or In vivo	Effects	Reference
Valproic acid + sphingosine-1-phosphate	In vitro	Enhanced migration, proliferation, colony forming units. Anti-inflammatory properties	Lim et al. [103]
Desferrioxamine (DFO)	In vitro	Low dose led to reduced mitochondrial activity and apoptosis of MSCs	Fujisawa et al. [97]
Dimethyloxalylglycine (DMOG)	In vitro In vivo	Increased cell survival and pro-angiogeneic factors (HIF-1α, VEGF). In vivo (rat myocardial infarction model) DMOG-MSCs reduced heart infarct size with improved therapeutic benefits	Liu et al. [101]
Anesthetic isoflurane	In vitro In vivo	Short low dose exposure enhanced MSC survival and migration. Upregulated HIF-1α, SDF-1, CXCR4. Activation of Akt similar to hypoxia treatment. In vivo (Rat middle cerebral artery occlusion model) isoflurane priming enhanced MSC engraftment in ischemic brain and improved outcome in mouse model of stroke	Sun et al. [100]
All-trans retinoic acid (ATRA)	In vitro In vivo	ATRA increased MSC expression of cell survival and growth factors (COX-2, HIF-1α, CXCR4, CCR2, VEGF, Ang-2, Ang-4). In vivo (rat wound healing model) ATRA primed MSCs were superior in wound closure with improved angiogenesis	Pourjafar et al. [99]
Rapamycin, everolimus, FK506 or cyclosporine A	In vitro In vivo	Immunosuppressant treated MSCs fivefold more suppressive of T-cell proliferation in vitro. MSCs adsorbed and released drugs to host cells in vitro. In vivo (humanised GvHD mouse model) low dose primed MSCs significantly inhibited onset of disease compared to untreated MSCs	Girdlestone et al. [104]
Adenosine receptor activation	In vitro	Activation of A1R (via 2-chloro-N6-cyclopentyl-adenosine (CCPA)) led to greater osteogenic differentiation via induction of osteogenic markers RUNX2 & alkaline phosphate (ALP) & mineralisation of extracellular matrix	D'Alimonte et al. [105]
Rapamycin	In vitro	Short but not long incubation with rapamycin enhanced MSC immunosuppressive effect. Effect mainly via upregulation of COX-2 and PGE2. mTOR inhibition significantly reduced IFN-γ induced MHC-II on MSC	Wang et al. [106]
2,4-dinitrophenol (DNP)	In vitro In vivo	In vitro enhanced expression of cardiomyogenesis, cell adhesion and angiogenesis genes. Intra-myocardial transplantation of DNP pre-conditioned MSCs led to enhanced adhesion to myocardial surface with more viable cells. Improvement in cardiac function, less scar formation, enhanced maintenance of left ventricular wall thickness and increased angiogenesis	Khan et al. [96]
All-trans retinoic acid (ATRA)	In vitro	ATRA treated MSCs in co-culture assays with AS patient’s PBMCs. Enhanced MSC IL-6 secretion. ATRA treated MSCs reduced Th17, T-regs, TNF-α, IFN-γ	Li et al. [102]
5-aza-2′-deoxycytidine (5-aza-dC)	In vitro	Methyltransferase inhibitor 5-aza-dC elevated endothelial markers (CD31, CD105, eNOS, VE-cadherin), promoted angiogenesis in matrigel assays. Upregulated endothelial differentiation inducers (VEGFA, ANGPT2, FGF2, FGF9 and ETS1)	Xu et al. [107]
All-trans retinoic acid (ATRA)	In vivo	In vivo (mouse elastase induced emphysema model) ATRA + MSCs combination increased static lung compliance, mean linear intercepts & alveolar surface area. P70S6 Kinase-1 overexpressing MSCs + ATRA even more beneficial. ATRA activated P70S6Kinase-1 enhanced accumulation and extended survival of MSCs	Takeda et al. [98]
β3 adrenergic agonists (β3AR)	In vivo	Agonist with CXCR4 antagonist mobilised MSCs to blood stream in rodents. Reversal of CXCL12 gradient across bone marrow endothelium and production of endocannabinoids. Significant induction of bone formation in rat spine fusion model	Fellous et al. [108]
Lithium + valproic acid	In vivo	Intranasal delivery of lithium and valproic acid treated MSCs in Huntington’s disease mouse model enhanced open field test, ambulatory distance and mean velocity. Benefits to motor function, reduced striatal neuronal loss and Huntington aggregates versus naïve MSCs. Increased MSC trophic effects, antioxidants, cytokine/chemokine receptors, migration, mitochondrial energy metabolism and stress response signaling pathways. Pre-treated MSCs survived longer after transplantation	Linares et al. [95]

Metabolic modifying and hypoxia mimetic agents such as 2,4-dinitrophenol (DNP) and desferrioxamine (DFO) are used to prime MSCs to enhance their survival and therapeutic effects [96, 97]. Fujisawa et al. [97] showed that low doses of deferoxamine (DFO, a hypoxia mimetic reagent) led to reduced mitochondrial activity and apoptosis in MSCs. When DFO conditioned MSCs were analysed for changes in metabolomic patterns, both hypoxia and DFO shared similar effects on MSCs [97]. 2,4-dinitrophenol (DNP) is another metabolic modifier, MSCs primed with DNP are shown to upregulate genes associated with cell adhesion, cardiomyogenesis and angiogenesis such as VEGF, CD90, CD44, CD29, serine/threonine-protein kinase (Kin)-3, atrial natriuretic peptide (ANP), connexin (C)-43, GATA binding protein (GATA)-4 and homeobox protein NKx 2.5. In vivo, DNP-primed MSCs led to improved survival, homing, adhesion, cardiomyogenesis and angiogenesis in a rat model of myocardial infarction. Intra-myocardial transplantation of DNP pre-conditioned MSCs led to enhanced adhesion of MSCs to the myocardial surface with more viable cells in comparison to normal MSCs leading to significant improvement in cardiac function with less scar formation, enhanced maintenance of left ventricular wall thickness and increased angiogenesis [96].

Inhibition of hypoxia-inducible factor 1α (HIF-α) prolyl hydroxylase via dimethyloxalylglycine (DMOG) has been used as another approach to prime MSCs to enhance their survival and therapeutic effects. DMOG priming is shown to enhance the expression of cell survival, pro-angiogenic factors (HIF-1α, VEGF). In in vivo (rat myocardial infarction model) DMOG-MSCs led to significant reduction in heart infarct size with improved therapeutic benefits in comparison to control MSCs [101]. Similar to hypoxia treatment, short term exposure of MSCs to the volatile anaesthetic isoflurane is shown to induce HIF-1α, stromal cell-derived factor (SDF)-1 receptor, CXCR4 and activator of protein kinase (Akt). In an in vivo rat middle cerebral artery occlusion model, isoflurane primed MSCs led to enhanced engraftment into the ischemic brain with improved therapeutic benefits [100].

MSCs are known to express adenosine receptors (A_1_, A_2A_, A_2B_, and A_3_). which are known to play a vital role in proliferation and differentiation of host cells. Activation of MSCs via adenosine 1 receptor agonist [2-chloro-N6-cyclopentyl-adenosine (CCPA)] was shown to induce enhanced osteogenic differentiation via induction of osteogenic markers RUNX family transcription factor (RUNX)-2 & alkaline phosphatase (ALP) and mineralisation of extracellular matrix. Mechanistically, CCPA were shown to activate wingless-related integration site (Wnt) signalling pathways, including the activation of dishevelled protein (DSh) while inhibiting glycogen synthase kinase 3b. Inhibition of dishevelled (Dsh) or Wnt signalling blockade interfered with CCPA mediated benefits from MSCs [105].

Immunosuppressive drugs such as rapamycin, everolimus, FK506 or cyclosporine A have been tested for their ability to boost the immunosuppressive properties of MSCs [104, 106]. Immunosuppressant treated MSCs are shown to be more suppressive in T-cell proliferation assay in vitro. Interestingly, this effect was due to the MSCs adsorbing the drugs and releasing them to the target cells [104]. Low dose or short term exposure of MSCs to rapamycin was shown to be beneficial in both in vitro and in vivo [104, 106]. In a humanised mouse model of graft versus host disease (GVHD), low dose rapamycin primed MSCs showed significant inhibition of the onset of the disease in comparison to the untreated MSCs [104].

Taken together, the studies discussed above support the priming strategy of MSCs via pharmacological drugs/small molecules with varying effects and potentially boosts their therapeutic benefits in a range of disease settings via improved survival, homing, engraftment and immuno-modulatory properties.

## Enhancement of extracellular vesicle production and function

The MSC secretome has been defined as the combination of soluble proteins, free nucleic acids, lipids and different types of extracellular vesicles (EVs) [107]. Utilising the MSC secretome, or secretome constituents, as a non-living therapeutic eliminates many of the risks and challenges associated with the use of living cell therapies [108]. Extracellular vesicles themselves can be further subdivided into apoptotic bodies, micro vesicles, and exosomes [109]. It is now widely reported that MSCs exert much of their paracrine and therapeutic potency through the release of EVs, which can range in size from 50 to 1000 nm in diameter [107, 110–112]. Secreted EVs have been proposed as a viable replacement for MSC therapy as they are loaded with many bioactive factors such as lipids, proteins (transcription factors, growth factors, and enzymes), nucleic acids (RNAs: mRNAs, microRNAs-miRNAs, and non-coding RNAs-lncRNAs; and DNA: ssDNA and dsDNA), and in some cases components of organelles (e.g., mitochondrial DNA) [113, 114].

Extracellular vesicles have been proposed as a viable replacement for MSC therapy with the additional benefit of being easier to produce and store. Preclinical studies have reported the potential of EVs as a therapeutic in liver [115] and cardiac [116] regenerative medicine. Bone marrow-derived MSC-EVs reduced brain cell death and improved neuronal survival and regeneration [117]. A recent clinical trial reported improved oxygenation and reduced cytokine storm in COVID-19 patients treated with EVs derived from BM MSCs [118]. Implementation of EVs for the treatment of many types of cancer has also shown to be a promising approach [119, 120].

There is an abundance of evidence to suggest that the composition of EVs in the MSC secretome is largely influenced by the environment to which the MSCs are exposed [111, 121]. Similarly EV production is scalable and production quality and quantity can be controlled by several factors [21, 111, 122]. Parameters such as MSC cellular confluence, cell passage number, oxygen availability and manipulating EV-biogenesis biology by priming with cytokines, heparin, and serum content of the medium can effect both EV quality and quantity [21, 112, 123]. Patel et al. [124] reported that MSC seeding density affected EV yield, with lower density being related to higher EV yields, while hypoxia has been shown to impact EV cargo [125]. Hypoxic preconditioning of MSCs has been shown to increase concentrations of VEGF, FGF-2, hepatocyte growth factor (HGF), insulin-like growth factor (IGF)-1 and TGF-β1 in CM [126–129]. Interestingly, preconditioning of MSCs with hypoxia increased the concentration of EV miRNA-21 and improved memory deficits in mice by restoration of synaptic function and regulation of inflammatory responses [130]. Considering that miR-21 can regulate cell survival by stimulating proliferation and by inhibiting apoptosis, this miRNA has been connected with MSC-EV-mediated therapeutic effects in various disease models [131]. In another study of subcutaneous fat grafting, hypoxia-preconditioned adipose MSC-derived exosomes were shown to promote angiogenesis and neovascularization as well as improved graft survival in a mouse model [132]. MSC-CM produced under hypoxic conditions has previously been shown to enhance wound closure in a preclinical skin injury model [127].

Priming with inflammatory cytokines such as IL-1β, TNF-α or IFN-γ was shown to initiate the production of immune-modulatory factors including granulocyte colony-stimulating factor (G-CSF), factor H, and galectins [129, 133, 134]. Ragni et al. reported that IFN-γ priming enhanced secretome anti-inflammatory potency [135].

Less is known about the capacity of priming to alter the EV content specifically, yet there is evidence to suggest that inflammatory preconditioning could help engineer specific MSC EVs. Inflammatory priming with TGF-β and IFN-γ cytokines promoted EVs to differentiate mononuclear cells into T-regs [136], while others report loss of EV protective function following inflammatory preconditioning [137]. De Jong et al. found that TNF-α changed the protein content of EVs from endothelial cells [125], while the same treatment improved adipose-derived exosome efficacy for bone regeneration [138]. Recently Cheng et al. investigated the effects of adipose-derived MSC priming with IFN-γ and TNF-α and reported upregulation of RAB27B, which increased the secretion of small EVs containing A20 and TNFα-stimulated gene-6 (TSG-6), key mediators of MSC immunopotency [114]. Conversely, a 2020 study reported that both IFN-γ and hypoxia priming of human BM-MSC had a minimal effect on EV miRNA content [139]. An in vitro study of IL-1β priming of MSC derived exosomes showed enhanced anti-inflammatory activity in osteoarthritic cells, concomitant with increased expression of anti-inflammatory factors (suppressor of cytokine signalling (SOCS)-3 and SOCS6), mediated by miR-147b and inhibition of the NF-κB pathway [140].

Preclinical studies have reported the potential of EVs as a therapeutic in liver [115] and cardiac [116] regenerative medicine. Bone marrow-derived MSC-EVs have been shown to reduce brain cell death and improved neuronal survival and regeneration [117]. A recent clinical trial reported improved oxygenation and reduced cytokine storm in COVID-19 patients treated with EVs derived from BM MSCs [118]. Implementation of EVs for the treatment of many types of cancer has also shown to be a promising approach [119, 120]. However, considering that the use of EVs as a potential stand-alone therapeutic is a relatively novel area of cell therapy, there is limited and sometimes conflicting data on how cell priming effects MSC-EV cargo and function. Priming appears to be a critical step that requires further investigation for deciphering the molecular mechanisms involved in EV biogenesis and recruitment of cargo and the subsequent production of EVs with a desired functional effect, particularly for the future use of EVs in clinical trials.

## Conclusions

It has become increasingly clear over recent years that, while stem cell therapy holds immense promise, the path to licensed medical options in the clinic remains difficult (see Table 3 for a brief overview of predicted advantages and concerns associated with each technique). Patient and disease variability, coupled with issues relating to manufacturing doses to scale, mean that the standard cell therapy product may not be the route to success. Here we have presented a range of enhancement strategies that may be able to push cell therapies into the realm of robust and reproducible benefit to the patient, with the promise of modifications to tailor therapies to specific diseases or even eventually to disease phenotypes. As the mechanism of action of the therapies are further elucidated we can also look forward to even more powerful improvements in efficacy using a host of techniques.

**Table 3:** Overview of modification techniques

Modification strategy	Advantages	Disadvantages
MSCs for drug delivery	Easily performed in culture	Short cell life after engraftment
	GMP grade existing drugs more readily available	Drug effects on stem cell itself
		Not possible to load all drugs
		Required short interval between loading and administration
Anti-microbial enhancement	Allows specific targeting of identified pathogens	Of less relevance outside infectious disease
Licensing strategies	Rely on endogenous mechanisms	Full licensing environment difficult to recreate
	Proven cryopreservation compatibility	
Hypoxia priming	Enhances cell proliferation during manufacture as well as efficacy	Requires specific manufacturing equipment
Differentiation prior to engraftment		Can be long and difficult manufacturing process
Gene modifications	A wide range of possible therapeutic proteins can be expressed	Unpredictable effects of transgene on stem cell
		Risk of stem cell mutagenesis with some vector options
Small molecule priming	Inexpensive and simple methodology	Possible off-target effects on MSC
	Wide availability of GMP compounds	
EV enhancement	Increased safety / lower immunogenicity due to no cell involved	Heterogeneity within EV batches due to culturing and isolation methods
	Easier storage and delivery of therapeutic	Large scale production still problematic

## Data Availability

All data is available on request.

## Abbreviations

MSC: Mesenchymal stromal cell
MHC: Major histocompatibility complex
hCNT1: Human concentrative nucleoside transporter 1
hENT1: Human equilibrative nucleoside transporter 1
P-gp: P-glycoprotein
MARCO: Macrophage receptor with collagenous structure
SR-B1: Scavenger receptor class B type 1
CD: Cell determinant
EV: Extracellular vesicles
CXCR4: Chemokine receptor type 4
GLUT: Glucose transporter
AMP: Anti-microbial peptides
LL-37: Cathelicidin antimicrobial peptidecathelicidin
PAMP: Pathogen-associated molecular patterns
LPS: Lipopolysaccharide
TNF: Tumor necrosis factor
BAL: Bronchoalveolar lavage
IFN: Interferon-γ
IL: Interleukin
CM: Conditioned media
IDO: Indoleamine 2,3-dioxygenase
BM: Bone marrow
HIF-1α: Hypoxia inducible factor 1α
ROS: Reactive oxygen specie
I/R: Ischemia/reperfusion
MAPK: Mitogen-associated protein kinase
NF: Nuclear factor
RILI: Radiation-induced lung injury
TGF: Transforming growth factor
AsAP: Ascorbic acid 2-phosphate
TRAIL: TNF-related apoptosis-inducing ligand
HSV-TK: Herpes simplex virus thymidine kinase
IV: Intravenous
CAR-T: Chimeric antigen receptor T-cell
SOX: Sex-determining region Y-type high-mobility-group box 9
TGF: Transforming growth factor
BMP: Bone morphogenetic protein
COL7A1: Collagen type VII alpha 1 chain
RDEB: Recessive dystrophic epidermolysis bullosa
CXCL4: Chemokine (C-X-C motif) ligand 4
ATRA: All-trans retinoic acid
DNP: 2,4-Dinitrophenol
b3AR: B3-adrenergic agonists
Th-17: T-helper-17
PBMC: Peripheral blood mononuclear cell
COX: Cyclooxygenase
CCR: C–C motif chemokine receptor
VEGF: Vascular endothelial growth factor
Ang: Angiopoietin
DFO: Desferrioxamine
Kin-3: Serine/threonine-protein kinase-3
ANP: Atrial natriuretic peptide
C-43: Connexin-43
GATA: GATA binding protein
Nkx 2.5: Homeobox protein 2.5
DMOG: Dimethyloxalylglycine
SDF: Stromal cell-derived factor
Akt: Activator of protein kinase
CCPA: 2-Chloro-N6-cyclopentyl-adenosine
RUNX: RUNX family transcription factor
ALP: Alkaline phosphatase
Wnt: Wingless-related integration site
DSh: Dishevelled
PGE: Prostaglabdin
mTOR: Mammalian target of rapamycin
FGF: Fibroblast growth factor
ETS1: Protein C-ets-1
HGF: Hepatocyte growth factor
IGF: Insulin-like growth factor
G-CSF: Granulocyte colony-stimulating factor
GMP: Good manufacturing practice
